# Effectiveness of a District-Level Comprehensive Acute Stroke Care Model for Management of Patients With Acute Stroke in Karnataka: Protocol for a Quasi-Experimental Study

**DOI:** 10.2196/93984

**Published:** 2026-08-14

**Authors:** Pradeep S Banandur, P R Srijithesh, Gautham Melur Sukumar, Arvind Banavaram, V S Binu, Nandakumar Dalavaikodihalli Nanjaiah, Santosh Loganathan, Thimappa Hegde, Komal Prasad C, Lavanya Garady, Aaheli Roy, Parama Basu, Deepika S Reddy, Arpitha Arun, Upashana Medhi

**Affiliations:** 1 National Institute of Mental Health and Neurosciences Bengaluru, Karnataka India; 2 Mazumdar Shaw Medical Centre Bengaluru, Karnataka India; 3 Narayana Institute of Neurosciences Bengaluru, Karnataka India; 4 Scientific Knowledge for Ageing and Neurological Ailments (SKAN) Research Trust Bengaluru, Karnataka India

**Keywords:** stroke care management, Comprehensive Acute Stroke Care Model, stroke CASCADE, stroke care model, stroke management protocol, health system assessment

## Abstract

**Background:**

Stroke is a leading cause of death and disability globally. India, like other low- and middle-income countries, faces rising stroke cases with significant regional variations and burden. Successful management of acute stroke requires timely recognition, quick transportation to a definitive health care facility, and specialized care for reducing mortality and morbidity. Stroke care in India is predominantly urban-oriented, private sector–dominant, with a shortage of trained or specialist personnel and limited access to infrastructure. There is a need to develop stroke care systems across various levels of health care delivery that are comprehensive and standardized for timely intervention, management, and referral.

**Objective:**

This study aims to develop and assess the effectiveness of a Comprehensive Acute Stroke Care Model (CASCADE) for managing patients who had an acute stroke in Karnataka (a state in South India) with a focus on improving survival, disability, and quality of life.

**Methods:**

This is a quasi-experimental preinterventional and postinterventional study design with the CASCADE as the intervention. This study will be conducted in 6 secondary and tertiary health care facilities, and the effectiveness of the model will be assessed on 510 patients who had a stroke for the first time (255 each in the preintervention and postintervention arms). Furthermore, the patients will be followed up on 28 days and the 3rd, 6th, and 12th month after the stroke to evaluate survival, disability, and quality of life before and after the intervention.

**Results:**

The study is funded by the SKAN Research Trust. Data collection for the preintervention arm has commenced and is currently ongoing. As of manuscript submission, approximately 100 cases have been recruited in the preintervention arm. Data analysis will begin after completion of recruitment.

**Conclusions:**

This unique, first-of-its-kind CASCADE encompasses triage algorithm, diagnosis, investigations, treatment, referral, and discharge interventions. An effective model will provide valuable insights into stroke management and is highly translatable into practice. The findings will have significant implications for policymaking and stroke-related public health programming in India.

**Trial Registration:**

Clinical Trials Registry-India CTRI/2023/04/051370; https://tinyurl.com/44wwehpr

**International Registered Report Identifier (IRRID):**

DERR1-10.2196/93984

## Introduction

### Background

Globally, stroke is the leading cause of disability and the second leading cause of death [1]. In the last two decades, the lifetime risk of stroke has increased by 50%, with 1 in 4 people estimated to have a stroke in their lifetime [2]. Annually, 6.5 million people die from stroke, constituting approximately 6% of deaths among those aged 15 to 45 years-. Over 143 million healthy life years are lost annually, with 15% lost among those aged 15 to 45 years. India, like other low- and middle-income countries, is experiencing a stroke epidemic. It is the fourth leading cause of death and fifth leading cause of disability in India [3]. Karnataka, a southern Indian state, ranks ninth among all states in India in incidence of stroke. With 109 new patients who had a stroke for every 1,00,000 of its population in 2019, Karnataka had higher incidence of stroke than the national average [4].

Appropriate, timely, and efficient stroke management is crucial for improved outcomes, as standardized care systems have shown to reduce mortality and enhance functional outcomes in acute stroke cases. Effective management requires prompt recognition, rapid transportation to health care facilities, and specialized care to minimize deaths and disabilities [5]. In India, stroke care is mostly urban centric and private sector driven, with shortages of trained specialists and limited access to imaging and diagnostics [6,7]. Even medical college hospitals face a shortage of neurologists, highlighting the need for alternative care models [8]. There is a critical need to develop comprehensive, standardized stroke care systems across different levels of health care delivery for timely intervention, management, and referrals [5,9-11]. While some care models exist, especially in hospital settings, community-based models tailored for India are rare [12].

### Aim

This quasi-experimental study aims to develop and assess the effectiveness of a Comprehensive Acute Stroke Care Model (CASCADE) in Karnataka, India, on improvement of preparedness, pathways to care, and management practices related to stroke in different health care settings and improvement in survival, disability, and quality of life (QoL) of patients who had an acute stroke.

## Methods

### Study Design

We plan to conduct a quasi-experimental preinterventional and postinterventional study.

### Study Setting

The health care facilities comprise 1 neurospecialty hospital (a caseload of approximately 300 cases per month) and 1 medical college hospital (a caseload of approximately 50 cases per month) at the tertiary level and 1 district hospital (a caseload of approximately 30 cases per month), 1 medical college hospital (a caseload of approximately 50 cases per month), and 2 government hospitals (a caseload of approximately 50 and 30 cases per month) at the secondary level in Karnataka.

### Characteristics of the Study Units

The study unit consists of health care facilities including health care personnel and patients who had a stroke. Health care facilities and their personnel will be the unit of study for stroke care preparedness, while all patients who had a stroke for the first time (both ischemic and hemorrhagic) attending the emergency rooms of these hospitals and/or admitted within the hospital during a 1-year period (or until the requisite sample size is obtained, whichever is earlier) will be the unit of study to assess CASCADE effectiveness before and after the intervention.

### Description of Materials

All aspects of stroke care from the time of onset to discharge and after discharge (for 1 year), namely preparedness, management, pathways to care, and the CASCADE, shall be assessed using semistructured, partially open-ended schedules (Table 1).

**Table 1:** Proposed content for each data collection time point in the schedule—Comprehensive Acute Stroke Care Model (CASCADE).

Name of the schedule	Contents or scale used	Study units	Time points of data collection
Preparedness schedule for a health care facility	Information related to all aspects of preparedness, including personnel, infrastructure, guidelines, training, and management related to stroke	Health care facilities identified for the CASCADE	Once in the preimplementation phase and once in the postimplementation phase
Baseline schedule for collecting basic information of patients who had a stroke to understand the existing management practices during the preimplementation phase and to assess the effectiveness of the developed CASCADE in the postimplementation phase	Detailed information on sociodemographic characteristics, risk factors, their date of onset or diagnosis, duration, dose or amount of risk factor as appropriate (eg, average number of cigarettes smoked per day and alcohol consumed per day), current status, and dependence information (as appropriate)	All patients who report to the hospitals enrolled for assessment of the CASCADE	As and when individuals report to study hospitals with a stroke during the study period (both preintervention and postintervention of the CASCADE)
Management schedule to understand the management of stroke cases	Includes case record forms with information related to history, clinical information, laboratory investigations conducted, management procedures performed, and drugs prescribed	All patients who report to the hospitals enrolled for assessment of the CASCADE	As and when individuals report to study hospitals with a stroke during the study period, preintervention and postintervention of the CASCADE
Pathways to care schedule to collect information on stroke outcome	Includes information related to different treatments and care sought in different places or systems of medicine before reporting to the study hospitals	All patients who report to the hospitals enrolled for assessment of the CASCADE	As and when individuals report to study hospitals with stroke during the study period, preintervention and postintervention of the CASCADE
Outcome schedule	Information related to survival, and disability: Modified Rankin Scale and Stroke-Specific Quality of Life Scale	All patients who report to the hospitals enrolled for assessment of the CASCADE	As and when individuals report to study hospitals with a stroke during the study period and subsequent follow-ups (28 days, 3 months, 6 months, and 12 months after the stroke)
CASCADE schedule	Information related to the standardized CASCADE. This shall contain treatment and management algorithms for stroke care at different levels of health care. This will be developed using information from the preparedness, management, pathways to care, and outcome schedules followed by validation by an expert advisory group. This will be a synthesis of data of all patients who report to the hospitals enrolled for assessment of the CASCADE in the preintervention phase.	All patients who had a stroke within the study hospitals who are eligible for assessment in both the preintervention and postintervention period of the study	As and when individuals report to study hospitals with stroke during the study period preintervention and postintervention of the CASCADE

### Process

#### Preintervention Phase

Initially, current stroke management practices at selected health care facilities are assessed to estimate their preparedness, including infrastructure, equipment, human resources, diagnostics, laboratories, and radiological investigations. Available drugs along with a gap analysis shall also be reviewed while assessing preparedness. A scoring system shall be developed based on preintervention inputs weighted by their importance in improving survival, reducing disability, and enhancing QoL. Specific indicators will be created from this scoring system to assess preparedness and management practices.

#### The CASCADE: Development, Implementation, and Assessment of Effectiveness

The intervention is the CASCADE, which will be developed through a process of observation and expert consultation. The CASCADE intervention is a structured, multicomponent health care facility–based intervention tailored to each participating facility based on findings from the hospital preparedness assessment and gap analysis. While site-specific modifications may differ according to identified gaps, the overall intervention framework will remain uniform across all participating health care facilities. The detailed intervention package will be developed using evidence generated from the preintervention stroke data, hospital preparedness assessments, Indian stroke management guidelines, and recommendations from an expert panel that will validate and standardize the protocols and algorithms across different levels of care. The proposed CASCADE will include procedures and standards for screening, triaging, and management of stroke cases at different levels of health care, algorithm-driven management, and referral of stroke cases. The effectiveness or outcomes of the CASCADE being assessed are stroke survival, disability, and QoL over a period of 1 year after the stroke.

**Figure 1:**
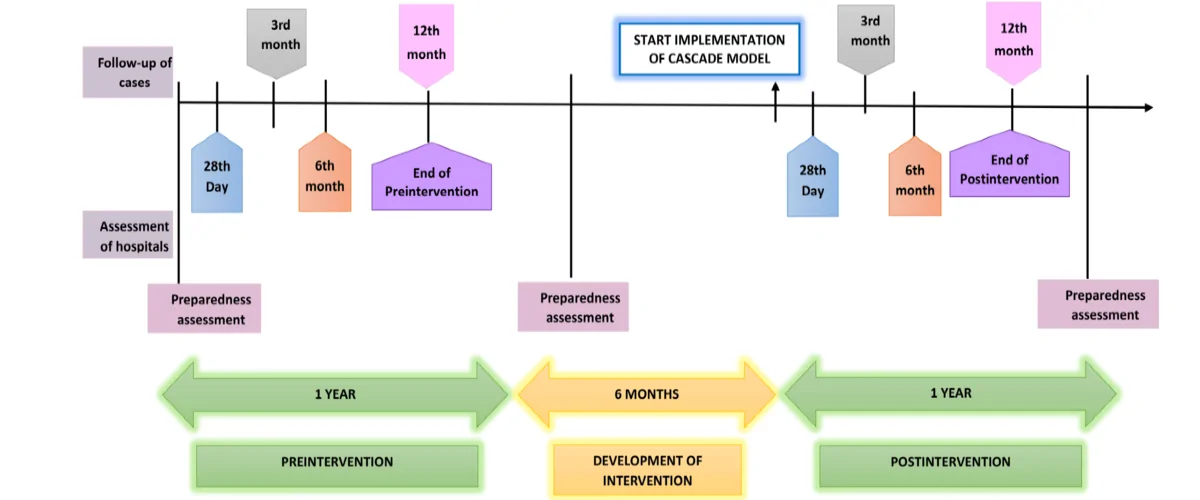
Schematic representation of the development and implementation of the Comprehensive Acute Stroke Care Model (CASCADE).

Once developed, the CASCADE will be implemented across study units. Health care facilities will be informed about their preparedness, management practices, and needed improvements based on preimplementation scores and indicators. Suggested modifications and personnel training will be provided. Training attendance, supportive supervision, and adherence to standardized protocols and algorithms will additionally be monitored during implementation. Implementation fidelity will be assessed using both facility-level and patient-level indicators. Preintervention and postintervention hospital preparedness assessments using standardized tools will evaluate changes in infrastructure, service availability, referral mechanisms, and stroke preparedness processes. The same indicators and scoring system will be used after the intervention to compare and estimate effectiveness of the CASCADE on outcomes of stroke care in the study unit. Figure 1 provides a schematic representation of development and implementation of the CASCADE.

### Data Collection

#### Patient-Related Information

Data collection will be conducted through face-to-face or telephonic interviews with patients who had a stroke or their caregivers (if patients are aphasic). Acute stroke cases will be assessed through an in-person interview at the time of presentation at hospital (recruitment), during the hospital stay, and at discharge. Clinical and laboratory records will be reviewed to collect data on stroke type, clinical presentation, investigations, patient and sample journeys, disability assessment, and treatment outcomes (death, disability, and QoL). Telephonic interviews are planned for follow-ups if travel is not feasible, with medical records sourced via email or other personalized social media platforms. This is being done to improve participant retention in the study.

#### Hospital Preparedness

Observation of facilities, record review, and interviewing the personnel in charge of the health care facility (key informant interviews) will be recorded.

Trained data collectors collect data using a low-literacy, user-friendly digital data collection platform. These data get uploaded into a password-protected (3 layers) server with access only to specific authorized individuals within the study team.

#### Follow-Up

After discharge, all cases will be followed up for a period of 1 year after the stroke at 4 time points (Figure 1): 28 days (for stroke-related deaths), 3 months (for disability), and 6 and 12 months (to assess the process of rehabilitation along with survival, disability, and QoL).

#### Monitoring and Review

The core team will closely supervise data collection with 5% repeat data collection for assessment of completeness, accuracy, and quality of data collected on patients. Any deviation or errors >10% would call for repeat assessment.

An independent monitoring team will oversee project implementation, comprising midlevel specialists from community medicine, neurology, and social work, ensuring adherence to protocol, conducts quarterly monitoring visits, and reports progress and challenges to the study team.

An expert advisory group, including specialists in neurology, neurosurgery, epidemiology, and public health, will provide strategic direction and technical support. They will also provide timely advice to investigators and review the master protocol, suggesting modifications as needed.

#### Training

The training for data collectors will be participatory, using different methods by specialized personnel for each domain. We plan to conduct induction and refresher training at the beginning of each data collection cycle to ensure standardization. To ensure quality apart from rigorous training, weekly and fortnightly review and problem-solving meetings will be held.

### Outcome Assessment

Increased survival, reduced disability, and improvement in stroke-specific QoL after introducing the CASCADE compared to the preintervention phase is considered an outcome. An increase in the scores and indicators of hospital preparedness and management practices is considered as the outcome for hospital preparedness.

### Study Duration

The study will be conducted for 4 years, which comprises of 6 months for development and implementation of the CASCADE and 1 year each for before and after the intervention.

### Sample Size

The primary patient-level outcome for sample size estimation is 28-day poststroke survival. Disability, QoL, rehabilitation processes, management practices, and hospital preparedness indicators are considered secondary outcomes and are assessed at the respective predefined follow-up periods. The sample size is estimated based on the Freedman formula [13] given below, where *n* is the total sample size required, *E* is the expected number of events in the study population, and and are proportion survived in group 1 (before intervention) and group 2 (after intervention), respectively, after 28 days of onset of stroke:

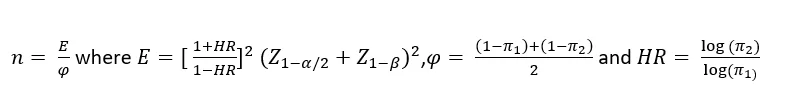



The expected 28 days’ survival percentage before the intervention is 42% (Sukumar et al, unpublished data, 2016). The estimated sample size for 80% and 90% power, expected percentage of survival after the intervention, and corresponding hazard ratios at a 5% level of significance is shown in Table 2.

**Table 2:** Expected percentage of survival after the intervention and corresponding hazard ratios at a 5% level of significance.

Expected proportion of survival after the intervention	Hazard ratio (group 2 or group 1)	Power 80%			Power 90%	
		Number of events (death among patients who has stroke) required	Total sample size	Number of events (death among patients who has stroke) required		Total sample size
0.52	0.75	398	752	536		1010
0.57	0.65	174	344	232		458
0.62	0.55	96	200	126		2641
0.67	0.46	60	130	80		176

A sample size of 510 (255 in each arm: preintervention and postintervention of the CASCADE) patients who had a stroke was fixed with an expected proportion of survival at 57% compared with 42% before introduction of the CASCADE. This sample size is expected to attain a power of 90% at a protective hazard ratio of 0.65, with an anticipated dropout rate of 10% at 28 days. The expected number of cases in a neurospecialty hospital is approximately 300 per month, in 2 medical college hospitals is approximately 50 cases per year each, in 1 government hospital is approximately 50 per year, and in 2 more government hospitals is approximately 30 per year. To ensure representation of all hospitals, we propose to enroll 50% of the patients from a neurospecialty hospital and the remaining 50% of patients from all other selected health care facilities.

### Statistical Analysis

The respondents in the preimplementation phase of the CASCADE are considered group 1 and those in the postimplementation phase as group 2. The primary outcome for CASCADE effectiveness includes improved hospital preparedness and improved patient treatment outcomes (death, disability, and QoL). The timing and sequence of implementation at each site will be documented in case of minor variations in the timing of intervention implementation across participating sites due to administrative or operational factors beyond the control of the investigators. These factors will be incorporated into the statistical analysis plan. Site-level and time-related variations will be adjusted for during analysis to account for potential differences in implementation timing across hospitals. We hypothesize that the treatment outcomes in group 2 will be better than those in group 1. The Kaplan-Meier method will be used for estimating the survival functions of the two groups at follow-up. The log-rank test will be used to compare the survival distributions between the two groups for each time point. Finally, the Cox proportional hazards regression analysis will be performed at each of the 4 time points separately in which the grouping variable is the intervention (CASCADE) in addition to potential confounders. Relevant patient-level covariates, including stroke severity, National Institutes of Health Stroke Scale score, timing of presentation, referral pathway, and other clinical characteristics, will be considered as potential confounders in adjusted analyses. The QoL before and after the implementation of the CASCADE will be compared using an independent sample 1-tailed *t* test or the Mann-Whitney *U* test depending on the normality assumption. Linear mixed models will be used in which QoL is the dependent variable and the CASCADE is the main independent variable. In the model, the group and the 4 time points are fixed factors and participants are considered as random. The modified Rankin Scale (mRS) scores before and after implementation of the CASCADE will be compared using the Mann-Whitney *U* test at each time point after adjusting for multiple comparisons using the Bonferroni method. Survival rate by different categories of stroke (hemorrhagic and ischemic) and by different severities of stroke (Glasgow Coma Scale and mRS based) will be used for evaluation of effectiveness of the model.

To address the possibility of time-varying confounding in the quasi-experimental study design, the study incorporates collection of several patient-level and system-level variables during both preintervention and postintervention phases. Patient-level variables include stroke severity, National Institutes of Health Stroke Scale score, mRS score, timing of presentation (including arrival within the golden hour), referral pathways, investigations performed, and treatments received. These variables will help assess differences in case mix and care processes across study periods.

In addition, hospital preparedness assessments conducted during both phases will capture system-level and organizational changes, including patient attendance patterns, staffing, infrastructure, referral mechanisms, service availability, and operational changes occurring over time.

These variables will be considered during analysis and interpretation to contextualize observed differences between preintervention and postintervention phases and reduce the impact of time-varying confounding. The objective is to achieve improved survival rate at every level of case severity in proportion to the level of the stroke care facility.

For improved hospital preparedness, the specific scoring system developed and indicator data will be compared using an independent samples *t* test or the Mann-Whitney *U* test depending on normality.

### Ethical Considerations

Ethics approval for this study was obtained from the institutional ethics committee of the National Institute of Mental Health and Neurosciences on June 17, 2022, #NIMHANS/35^th^ IEC (BS&NS DIV.)/2022. This trial was registered under the Clinical Trials Registry-India (CTRI/2023/04/051370) April 6, 2023. Any protocol modifications will be reported to the ethics committee and the trial registry. Administrative approvals were obtained from all the participating hospitals. Informed consent will be sought from the patient or caregiver ensuring priority for care, privacy, and confidentiality. Informed consent will be administered either in the local language (Kannada) or English as preferred by the respondents. Illiterate individuals will have the consent form read to them in the presence of a literate witness and will be requested to provide a thumb impression. In cases where the patients are aphasic, unconscious, or otherwise unable to provide informed consent due to severe disability or impaired decision-making capacity, written informed consent will be obtained from the primary caregiver or legally acceptable representative accompanying the patient in the presence of a witness. The electronic data collection system includes a field to identify whether the respondent is the participant or the caregiver. Where caregiver consent is obtained, the caregiver’s relationship with the participant will also be recorded. Wherever feasible, if the participant subsequently regains communication ability or decision-making capacity, the study procedures will be explained directly to the participant and their consent or assent for continued participation will be obtained.

### Dissemination

We plan to communicate to the participants and general public, scientific audiences, and policymakers about the results of the trial in various platforms including mandatory reporting to the trial registry, publishing in scientific journals, and providing a plain language summary as well as fact sheets.

## Results

Data collection for the preintervention phase has started from June 2025 and is ongoing. As of February 2026, 98 patients have been recruited out of 255 planned as per sample size calculation. Recruitment is continuing and is to be completed by June 2026.

## Discussion

### Principal Findings

This first-of-its-kind endeavor, the CASCADE seeks to reduce stroke-related mortality and morbidity while improving the long-term survival of patients with QoL for both the patients and their caretakers. This protocol aims at estimating current management practices and pathways to care and to evaluate the effectiveness of the developed CASCADE, which includes triage algorithms, diagnosis, investigations, treatment, referral, discharge-related interventions, and an assessment of health care facility preparedness for acute stroke care across different levels of health care. The outcomes assessed are improvement in survival, disability, and QoL of patients who had an acute stroke.

The proposed CASCADE is more comprehensive and encompasses the full continuum of care for stroke across various levels of health care. Most studies assess either one or few components of care during a hospital stay such as a critical path for nursing care, an emergency room management algorithm, and a hospital unit physician’s order sheet [14]. Very few studies have looked at follow-up care [15,16]. As far as we understand, there are no studies conducted that assess the quality of stroke care, which includes infrastructure, imaging facilities, or readily available stroke care units. This assumes importance along with the lack of availability of specialists at different levels of health care. There are only 2300 neurologists for a population of 1.4 billion, and they are mostly urban centric. Adding to this, we have an immensely skewed health care system along with radiological services. Furthermore, the lack of organized stroke care networks in urban areas makes effective acute stroke intervention difficult. The CASCADE shows promise by including the full continuum of care from the time of occurrence of stroke until 1 year after the stroke across different levels of health care. This includes pathways of care prior to admission, in-hospital triage, care and management practices, patient and sample journeys within health care settings, and posthospitalization care.

Other options to achieve the objectives were considered before deciding on the quasi-experimental study design. Stroke registries are a known resource for developing stroke care models [17] but lack the comprehensiveness required for our study due to limitations such as nonuniform data collection; method used; and unreliable information on management practices, care pathways, and rehabilitation. These limitations make stroke registries unsuitable for our objectives. Given the objectives, a cluster randomized trial or stepped-wedge design could have been a more appropriate design. It is known that the cluster or stepped-wedge designs are suitable for implementation on a large scale [18]. In our study, patients who had a stroke are recruited from a limited number of 6 strategically selected health facilities in Bengaluru and Kolar. These facilities were chosen based on patient admission trends and existing referral pathways. Considering this and the practical feasibility in evaluating the model in real-world settings, a quasi-experimental study design was chosen. Quasi-experimental predesign and postdesign may be influenced by time trends and other external factors. However, the effect of changes in stroke systems of care itself is one of the key aspects being evaluated in this study. Changes in survival, disability, and QoL may be influenced by hospital-level factors, patient case mix, referral patterns, staffing, diagnostics, and organizational processes. Several of these changes may occur because of gaps identified during baseline assessments and may therefore become part of the intervention exposure itself rather than only representing a source of bias.

At the same time, factors that are not directly related to the intervention but are likely to change during the study period will be documented and considered during analysis. A separate hospital preparedness assessment tool will be administered during both preintervention and postintervention phases to capture facility-level and system-level factors, including patient attendance, infrastructure, staffing, service availability, referral mechanisms, diagnostics, and other organizational changes. These data will help contextualize observed improvements and assess whether changes may be related to broader system-level modifications occurring alongside the intervention.

### Strengths

This study has several strengths. First, the model includes both individual-level and macrolevel interventions. Unlike existing models, which are primarily designed for tertiary care settings, the CASCADE is being developed for implementation across various health care levels, incorporating algorithm-driven management and referral of stroke cases. Second, the model addresses the entire continuum of care, from the onset of stroke to posthospital care, focusing on survival, disability, and QoL for 1 year after the stroke. Third, it includes comprehensive assessments of both prehospital and posthospital care pathways, which is a unique strength. Additionally, the study evaluates within-hospital patient journeys, acknowledging their impact on treatment outcomes.

Allocation of sample size is based on the caseload in each of the identified hospitals. Quality of data collection and processes are monitored by the independent monitoring team members and the expert advisory group. This will validate applicability and results of the CASCADE.

### Limitations

This study has certain limitations that need mention. First, the effectiveness of the CASCADE will be assessed in only a few settings in Kolar and Bengaluru, which might not be representative of all the settings within Kolar and Bengaluru. However, the model developed would be replicable (with appropriate modifications to suit local needs), given the uniform presentation of stroke cases and the hospitals selected largely represent hospitals similar to those in Bengaluru and Kolar.

Second, being a quasi-experimental study, there might be some residual confounding for the effect measures assessed due to unknown confounders. Given the inability to incorporate randomization into the study design, this limitation is unlikely to be completely negated. Furthermore, the current methodology adopted is likely to provide valuable insights into how the CASCADE might work in real-world situations where confounders cannot be completely controlled. Finally, this being a multisite study, it is likely that coordination and cooperation of study sites become a challenge. The study team and the institution have a strong working relationship over the years with these hospitals. The study institution also has a significant presence in the field, with Kolar being their designated public health observatory.

### Policy Implications

If effective, the CASCADE is likely to be adopted widely. Developed through a robust methodology, the preimplementation phase identifies current care practices and gaps, guiding the model’s refinement. Finalized with expert and stakeholder input, the intervention phase will assess the model’s impact on survival, disability, and mortality. Given its evidence-based development and validation, the model is replicable and could become standard practice in India and other low- and middle-income countries.

## Data Availability

This work was supported by the SKAN Research Trust.

## Abbreviations

CASCADE: Comprehensive Acute Stroke Care Model
mRS: modified Rankin Scale
QoL: quality of life
